# Thickness Effect on the Solid-State Reaction of a Ni/GaAs System

**DOI:** 10.3390/nano12152633

**Published:** 2022-07-30

**Authors:** Selma Rabhi, Nouredine Oueldna, Carine Perrin-Pellegrino, Alain Portavoce, Karol Kalna, Mohamed Cherif Benoudia, Khalid Hoummada

**Affiliations:** 1 IM2NP, CNRS UMR 7334, Aix-Marseille University, 13397 Marseille, France; selma.rabhi@ensmm-annaba.dz (S.R.); carine.perrin-pellegrino@im2np.fr (C.P.-P.); alain.portavoce@im2np.fr (A.P.); khalid.hoummada@im2np.fr (K.H.); 2 L3M, Ecole Nationale Supérieure des Mines et de la Métallurgie, Annaba, Sidi Amar 23000, Algeria; mohamed-cherif.benoudia@ensmm-annaba.dz; 3 Nanoelectronics Devices Computational Group, Swansea University, Swansea SA1 8EN, UK; k.kalna@swansea.ac.uk

**Keywords:** solid-state reaction, thickness, Ni-thin films, III-IV semi-conductors, in situ X-ray diffraction, intermetallic growth

## Abstract

Ni thin films with different thicknesses were grown on a GaAs substrate using the magnetron sputtering technique followed by in situ X-ray diffraction (XRD) annealing in order to study the solid-state reaction between Ni and GaAs substrate. The thickness dependence on the formation of the intermetallic phases was investigated using in situ and ex situ XRD, pole figures, and atom probe tomography (APT). The results indicate that the 20 nm-thick Ni film exhibits an epitaxial relation with the GaAs substrate, which is (001) Ni//(001) GaAs and [111] Ni//[110] GaAs after deposition. Increasing the film’s thickness results in a change of the Ni film’s texture. This difference has an impact on the formation temperature of Ni_3_GaAs. This temperature decreases simultaneously with the thickness increase. This is due to the coherent/incoherent nature of the initial Ni/GaAs interface. The Ni_3_GaAs phase decomposes into the binary and ternary compounds xNiAs and Ni_3−x_GaAs_1−x_ at about 400 °C. Similarly to Ni_3_GaAs, the decomposition temperature of the second phase also depends on the initial thickness of the Ni layer.

## 1. Introduction

With the exponential development of photonics, optoelectronics, photovoltaics, and microelectronics, the attention of researchers has turned to new materials that are more efficient than silicon, such as III-V semiconductors [1,2,3,4,5,6,7]. III-V semiconductors have promising physical and electrical properties such as direct band gap and high charge carrier mobility [8,9,10,11]. Their on-chip integration with a Si technology delivers enhanced functionality and performance while reducing the manufacturing costs [12,13,14,15,16]. Indeed, Si-based microelectronics has reached physical limits when it comes to nanoscale devices; the III-V materials and especially InGaAs appear as an appealing alternative for next-generation devices such as terahertz (THz) [17] and quantum computing applications [18]. InGaAs MOSFETs were recently shown exhibiting a promising mobility of 1100 cm^2^/V·s, significantly higher than that of state-of-the-art Si MOSFETs [19]. Furthermore, InGaAs offers high data rate transfer and high performance communication for photonic application [20] and is particularly appealing for photovoltaic applications, as it can theoretically provide a maximum efficiency of 49% [21]. However, to achieve a fully functional device, it is crucial to improve the metal/semiconductor contact issues. Ni proved to be a promising option for InGaAs as a contact material because it leads to a reliable ohmic contact, which is a major advantage [22,23]. In this work, we chose the Ni/GaAs metal-semiconductor system to understand the different phenomena that may occur upon the various fabrication processes since the GaAs substrates are similar to InGaAs processes due to the complete miscibility of In and Ga in the zinc-blende structure [24,25]. This is why the understanding of phenomena involved in both Ni/GaAs [26] and Ni/InAs [27] solid-state reactions is crucial for the fabrication and development of components for optoelectronics, photovoltaics, and microelectronics based on III-V semiconductors.

The solid-state reaction between metal films and GaAs substrates has been conducted to achieve intermetallic compounds with good and stable contact [28,29,30,31]. The solid state reaction between Ni film and GaAs substrate leads to the epitaxial formation of the intermetallic Ni_3_GaAs phase [26,32,33,34,35]. The growth of the phases is coupled and depends on the mass-balance at the interfaces, which itself depends on the diffusion of Ni, Ga, and As species. This step requires a good control of the phases’ formation, their kinetics, and the involved phenomena to provide a global and predictive response to the realization of the devices. The characteristics of the reactions occurring in thin films such as kinetics, sequence, etc., are different from the case of bulk materials. The differences are related to the thickness of the deposited metal [36] and the presence of numerous interfaces compared to the volume of the films considered [37,38]. For that reason, the investigation of the thickness effect of the intermetallic phase formation is crucial. Aside from electrical performances, it is also necessary to understand the so-formed material in terms of phase sequence, thermodynamics, and texture. The control of the phase formation and uniformity of the intermetallic interface is essential in the case of thin films. The formed phase should be in metastable equilibrium with the substrate to guarantee a stable contact.

Hence in this work, the solid-state reactions between 20, 100, and 500 nm-thick Ni films and GaAs substrate have been investigated using the state-of-the-art characterization techniques. In situ and ex situ X-ray diffraction (XRD) and pole figures have been carried out to characterize the initial state, texture, and sequence of the films’ phase formation. Through atom probe tomography (APT), the stoichiometry and the homogeneity of the so-formed intermetallic phase were defined.

## 2. Materials and Methods

Three different thicknesses (20, 100, and 500 nm) of Ni metal were deposited on GaAs (100) wafers in a commercial magnetron sputtering system exhibiting a base pressure of 10^−8^ Torr. All the samples were deposited at room temperature using a 99.9999% pure Ar gas flow (5 × 10^−3^ mbar) to sputter a 300 mm-wide 99.99% pure Ni target purchased from CODEX international. The sputtering power of the Ni target was 150 W. The sputtering is more suitable than molecular beam epitaxy (MBE) for mass-production applications [39]. The GaAs (100) substrate was cleaned with diluted nitric acid (HNO_3_) before Ni deposition. The Ni layer was capped with a 20-nm-thick layer of TiN to protect the samples from atmosphere contamination. The capping process was performed in the same setup as Ni deposition and the samples were transferred from one chamber to another without breaking vacuum. The thickness of the Ni and TiN layers for both the 20- and 100-nm-thick samples were confirmed by X-ray reflectivity and Transmission electron microscopy (TEM). The thickness of the 500 nm-thick sample was checked using scanning electron microscopy (SEM) in the cross-sectional mode. The structure of the as-deposited films was identified by X-ray diffraction (XRD) in the Bragg-Brentano geometry in a two-circle PANalytical X’Pert MPD-PRO setup equipped with an X’Celerator detector designed for high-speed data collection, using a Cu K_α_ source (λK_α_ = 0.15418 nm). In addition, to make pole figures on the 20- and 100-nm-thick Ni samples, the samples were characterized using the X’Pert MRD-PRO diffractometer with four circles to obtain the projection of the intensity I2θ (φ, ψ) in a two-dimensional form. The pole figure simulations and drawings of crystal structures were carried out using the CaRIne Crystallography software [40]. The cross-sectional SEM images were acquired using a Helios NanoLab DualBeam Ga+ focus ion beam (FIB) from FEI. For in situ XRD measurements, the samples (20-, 100-, and 500-nm-thick layer of Ni) were mounted in an Anton-Paar TTK600 temperature chamber equipped with a heating stage under vacuum (10^−6^ Torr) from 50 °C to 400 °C using a step of 10 °C with a 5 °C·min^−1^ ramp. For each step, a diffractogram was recorded for 8 min. The Atom Probe Tomography technique was used to analyze the atomic distribution in three dimensions of the 500-nm-thick layer annealed at 200 °C for 1 h. A specific specimen preparation was realized with the focused ion beam (FIB) standard lift-out technique. In this paper, a low-energy ion beam of 2–5 keV was used to clean the top of the sample at the final step of the specimen preparation. APT analyses were carried out in a LEAP 3000X HR instrument. The laser pulsing rate was set to 200 kHz, and the detection rate was kept to 0.002 event/pulse by increasing the applied voltage. During the analysis, the specimen temperature was set to 54 K. The IVAS 3.6.8 software was used for the reconstruction, visualization, and analyses of the APT data.

## 3. Results

**Figure 1:**
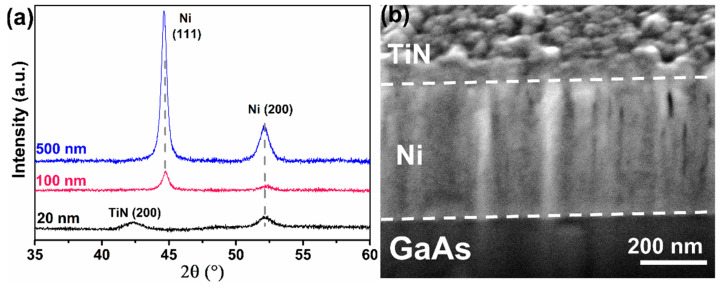
(**a**) Ex situ XRD diagrams for the as deposited Ni/GaAs samples with different Ni layer thicknesses (20 nm, 100 nm, and 500 nm) and (**b**) cross-section SEM image of the as-deposited 500 nm-thick sample.

The solid-state reaction that leads to the formation of an intermetallic phase in the Ni/GaAs system depends on the initial state of the as-deposited samples. Figure 1a shows the XRD signals measured for three samples with different Ni thicknesses (20, 100, and 500 nm). For the 100- and 500-nm-thick films, we observed two peaks at 44° and 52°, which correspond to (111) and (200) planes of the fcc structure of Ni. For the 20-nm-thick Ni film, only the (200) peak at 52° was observed, while the intense peak (111) of the Ni phase was not recognized. We can conclude that the 20-nm Ni sample shows a different texture in comparison to the thicker samples. The (200) orientation is favored at low thicknesses that corresponds to the orientation of the GaAs substrate. In addition, for the same sample, a new peak is recorded at 42°, corresponding to the (200) plane of TiN cubic structure; this is probably related to the texture of the 20-nm-thick Ni film. As we have mentioned earlier, all the samples were protected by a 20-nm-thick TiN layer in order to see the interface morphology present in the sample. Figure 1b shows the cross section of the 500-nm-thick Ni sample. We can see that the interface between Ni and GaAs is homogeneous, and that the thickness of the Ni layer is 512 ± 2 nm.

**Figure 2:**
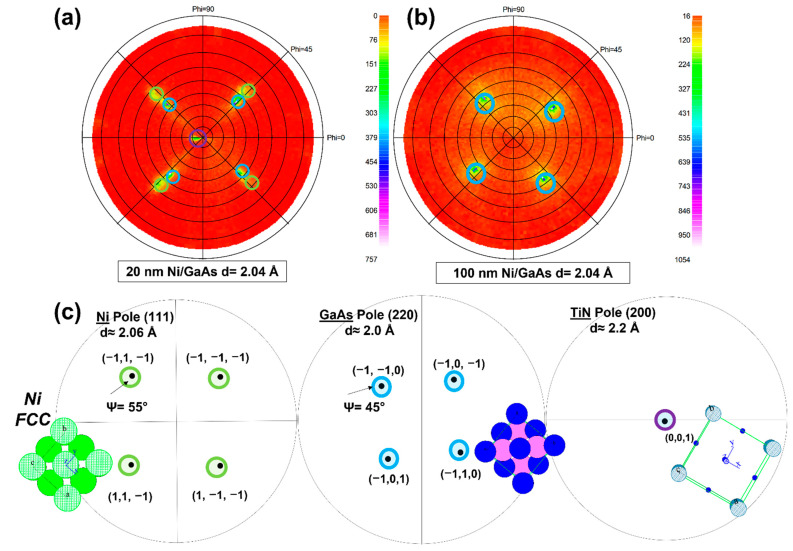
Experimental pole figure around the Ni (111) position at d = 2.04 Å for (**a**) 20-nm-thick Ni and (**b**) 100-nm-thick Ni samples after deposition on GaAs and (**c**) simulation of the pole figures of: Ni FCC, TiN, and GaAs substrate.

Figure 2a,b shows the pole figures of the as-deposited 20- and 100-nm-thick Ni layers. The pole figure was performed at the angular position of the Ni peak at d = 2.04 Å corresponding to the (111) crystallographic plane. The angular ranges probed were φ [0°, 360°] and ψ [0°, 84°]. The intensity I is represented as a function of φ and ψ, and it is characterized by the color nuances going from red to purple for the weakest to the strongest intensities, respectively. The concentric circles on the diagram define the angles ψ with a step of 10°. Figure 2c presents a schematic diagram of diffraction patterns simulated for the capping layer TiN, the substrate GaAs, and the metal layer fcc-Ni, with the lattice parameter a = 3.52 Å. Figure 2a shows, for the 2-nm-thick Ni sample, four poles located at ψ ≈ 45° and φ ≈ 46°, 136°, 226°, and 316° corresponding to the poles (110) of GaAs substrate (blue circle); right above these poles, four other less intense poles are located at ψ ≈ 55° (with φ ≈ 46°, 136°, 226°, and 316°) corresponding to (111) poles of fcc-Ni structure (green circle), and the pole in the center corresponding to (200) pole of the TiN layer. However, Figure 2b shows the pole figure measured for the 100-nm-thick Ni. Only the (110) poles of the GaAs substrate at ψ ≈ 45° and φ ≈ 43°, 132°, 223°, and 312° were observed. As a result, the grains of the Ni are randomly oriented, which is a sign of the absence of epitaxial relation with the substrate for the thick films. This is in agreement with the XRD results. The same result was observed for the 500-nm-thick sample.

**Figure 3:**
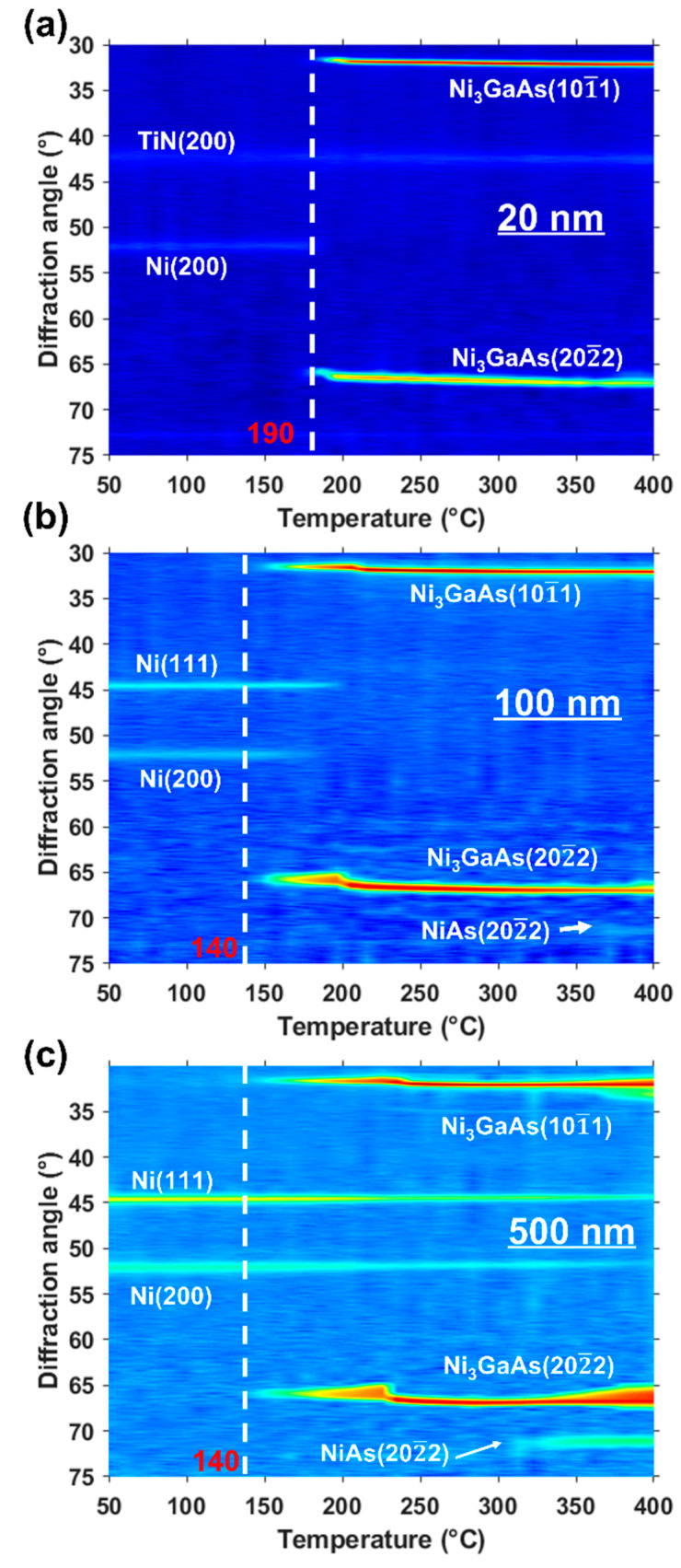
In situ XRD diagrams (λ = 1.5418 Å) recorded for: (**a**) 20 nm; (**b**) 100 nm; and (**c**) 500 nm Ni on GaAs (100) during an annealing between 50 °C and 400 °C.

In situ XRD characterization described below shows the evolution of the first phase ‘Ni_3_GaAs’ after a solid-state reaction between Ni film (with different thicknesses) and GaAs (100) substrate. The overall goal was to understand the thickness effect on the phase formation under annealing. Figure 3 illustrates the XRD results for the three samples presented as an intensity color map. The y-axis carries the 2θ angle, the x-axis carries the annealing temperature, and the intensity is represented by a color level code. The as-deposited 20-nm-thick Ni film already presents three diffraction peaks at RT, one at 2θ = 52° corresponding to Ni (200), and two at 2θ = 42° and 73° corresponding respectively to the atomic planes (200) and (311) of the TiN phase (Figure 3a). From 190 °C, the Ni (200) peak disappears, and two other peaks appear at 2θ = 31.6° and 65.9°. These peaks belong to the ternary phase (Ni_3_GaAs) that consumed the Ni layer and correspond to the atomic planes (10$\bar{\;1}$1) and (20$\bar{\;2}$2) of Ni_3_GaAs [35]. Figure 3b,c shows the 100-nm- and the 500-nm-thick Ni samples, respectively. As shown below, only two diffraction peaks are observed at 2θ = 44.6° and 52.1° corresponding to the atomic planes (111) and (200) of the fcc-structure of Ni, respectively. For the 100-nm-thick sample, the Ni peaks disappear around 200 °C unlike the 500-nm-thick sample, where Ni is present until the end of annealing. At 140 °C, two peaks appear at 2θ = 31.6° and 65.9° that correspond to the atomic planes (10$\bar{\;1}$1) and (20$\bar{\;2}$2) of Ni_3_GaAs, respectively. For the 100- and 500-nm-thick samples, above the 360 °C and 330 °C temperatures, respectively, we observe the appearance of one peak at 71°, which probably corresponds to the atomic plan (20$\bar{\;2}2$) of the binary phase NiAs [41].

**Figure 4:**
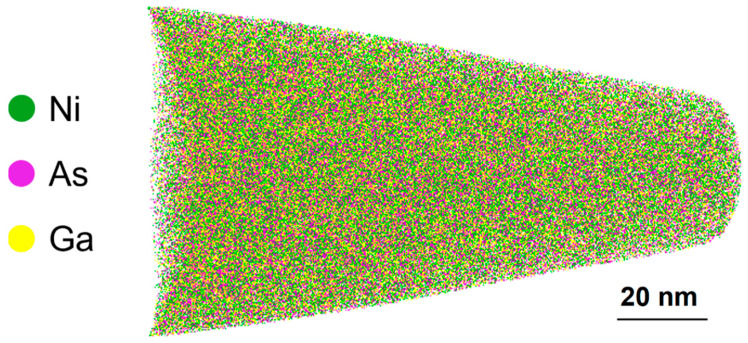
APT microscopy performed on the 500 nm Ni/GaAs after an isothermal annealing at 200 °C, Cross-sectional view of a 10 nm-thick slice (xz plane) extracted from 3D volume showing the distribution of Ni, Ga, and As atoms (green, yellow, and pink dots, respectively).

Figure 4 presents the APT measurements performed on the 500-nm-thick sample annealed at 200 °C for 1 h in order to quantitatively measure the chemical composition of the formed phase. As expected, a homogenous distribution of all atoms is observed, suggesting the presence of a single phase; no impurities were observed that could be beneficial to the electrical properties. The composition is deduced from the mass spectrum, in which no mass overlaps were found. The chemical composition averaged over the whole volume was 61.1% Ni, 17.7% Ga, and 21.2% As, indicating that the initial proportions of Ga and As in the substrate are almost kept in the so-formed phase. The composition corresponds to Ni_3.06_Ga_0.89_As_1.06_, which confirms the formation of the Ni_3_GaAs as the first phase for the thickest sample. This is in good agreement with the stoichiometry found for the 20-nm-thick Ni film [26].

**Figure 5:**
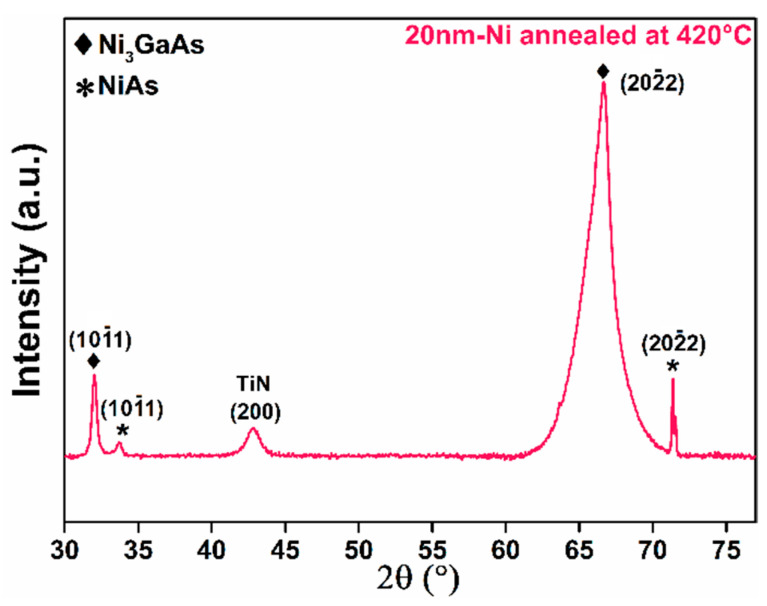
Ex situ XRD diffractogram for 20-nm-thick Ni sample annealed at 420 °C.

The second phase is observed for the 100- and 500-nm-thick Ni samples through the presence of a diffraction peak at 2θ = 71°. This peak appears at about 360 °C for the 100-nm-thick Ni sample (Figure 3b) and at 330 °C for the 500-nm-thick sample (Figure 3c). In contrast, no peak was observed for the second phase for the 20-nm-thick sample (Figure 3a). We assumed that for the 20-nm-thick Ni, the formation temperature was probably higher than 400 °C (Figure 3). For this purpose, the 20-nm-thick Ni was annealed up to 420 °C for 2 h in a vacuum oven. The ex situ XRD measurement is presented in Figure 5. Two new peaks have been observed at 2θ = 33° and 71°, corresponding to the atomic planes (10$\bar{\;1}$1) and (20$\bar{\;2}$2) of the NiAs phase, respectively [41] (Figure 5).

## 4. Discussion

The ex situ XRD and pole figure measurements showed that the texture of Ni differs with the film thickness increasing. Thus, Ni in the three samples has a fcc structure. Our findings are different from the previous published work, where the Ni film in a range from 1 to 40 nm thickness exists under two forms. The bcc-Ni structure can be indeed epitaxially grown on a GaAs (001) substrate at room temperature up to 2.5 nm [39,42,43,44,45]. However, this bcc structure is metastable. While the thickness is increased (beyond 2.5 nm), the bcc-Ni crystal starts to transform into a more stable fcc-Ni structure. The fcc-Ni is always epitaxial with the GaAs substrate until 40 nm of Ni [39,42]. However, Figure 2 shows that Ni film exists only in the fcc structure in both 20- and 100-nm-thick samples. There is no sign of the bcc structure. For the 20-nm-thick Ni, the Ni layer is in epitaxy with the GaAs substrate; the epitaxy relationship between the Ni film and the GaAs substrate is: (001) Ni//(001) GaAs and [111] Ni//[110] GaAs (Figure 2). TEM observations used to measure the thickness of the different layers show that the 20-nm-thick TiN layer is polycrystalline on all the samples. However, TiN diffraction is in general not detected in our case, probably due to a TiN texture not compatible with our diffraction conditions (Bragg-Brentano geometry and 35° to 60° diffraction window). The sample with the thinner 20-nm-thick Ni film is an exception. Indeed, a single TiN diffraction peak is detected at 42° corresponding to the (200) atomic planes of the TiN fcc structure (Figure 1a). Since the relation of epitaxy between Ni and GaAs, favoring the alignment of the fcc-Ni (200) planes parallel to the surface, is only observed for this sample, the specific texture of the TiN grains in this sample seems to be related to the specific texture of Ni. The 20-nm-thick Ni film is epitaxial on GaAs; however, as the film became thicker (≥100 nm), the grains were oriented randomly. We observed that the pole figure for the 500-nm-thick layer was identical to the 100-nm-thick Ni since they show the same XRD results (Figure 1). There seems to be a transition thickness over which the Ni layer can no longer grow in epitaxy, between 40 and 100 nm.

In situ XRD and APT analysis shows that the first phase to form during the solid-state reaction between Ni film and GaAs substrate is Ni_3_GaAs and not Ni_2_GaAs [26], in contrary to what was reported in literature [34,46,47,48]. The epitaxial relationship between this phase and the GaAs substrate was also evidenced: (111) GaAs//(0001) Ni_3_GaAs and [1$\bar{1}$0] GaAs//[11$\bar{2}$0] Ni_3_GaAs [26]. This relationship was first determined for the same thin film system [34] and, recently, for the intermetallic phase Ni_3_In_0.53_Ga_0.47_As [49]. As the diffraction peaks according to our measurements are the same, we can assume that the identification of the first phase is the same for all the samples and that the growth is epitaxial with the same relationship.

With a stoichiometry of Ni_3_GaAs, Ni should be the main diffusing species, as the Ga and As proportions are equivalent in the GaAs substrate [49]. Before the Ni consumption ends (Figure 3), Ni_3_GaAs exhibits a composition gradient based on two thermodynamic equilibria (Ni/Ni_3_GaAs and Ni_3_GaAs/GaAs) between the Ni film and the GaAs substrate. When the Ni film is completely consumed for the 20- and 100-nm-thick samples (Figure 3a,b) or blocked for the 500-nm-thick sample (Figure 3c), the system evolves towards a single equilibrium between Ni_3_GaAs and GaAs, resulting in the homogenization of the Ni_3_GaAs composition [28]. This homogenization induces a change of composition, and as a result, a shift of the diffraction peaks towards high angles [27,49].

The formation temperature of the Ni_3_GaAs phase was found to be different between the 20-nm-thick Ni sample and the two thicker 100-nm and 500-nm samples (Figure 3). It drops down from 190 °C for 20-nm-thick Ni film to 140 °C for 100- and 500-nm-thick Ni films (∆T ≈ 50 °C). Thus, the phase needs more energy to form (at 190 °C) for the 20-nm-thick sample in comparison to the two other samples for which the same phase is formed at a lower temperature (140 °C). This can be explained by the nature of the initial germination interface of the phase. As we have seen, the texture of Ni is not the same between small thicknesses and large thicknesses, and to germinate a phase at the Ni/GaAs interface, a germination barrier ∆G*, which is related to the interface energy γ, must be exceeded:

(1) $$∆G^{*}=\frac{16\pi ∆\gamma^{3}}{3\left(∆G_{V}-∆G_{S}\right)}$$

where (∆G_V_ − ∆G_S_) is the driving force linked to the formation of the same phase. ∆γ is the difference between the interface energy after phase formation and the initial interface energy, given by:

(2) $$∆\gamma =\left(\underset{\gamma \;\mathrm{after}\;\mathrm{formation}}{\underbrace{(\gamma_{\mathrm{phase}/\mathrm{GaAs}}+\gamma_{\mathrm{phase}/\mathrm{Ni}}}}\right)-\underset{\mathrm{initial}\;\mathrm{interface}}{\underbrace{\gamma_{\mathrm{Ni}/\mathrm{GaAs}}}}$$

The energy of a coherent interface is known to be lower than the energy of the incoherent one γ_epitaxy_ << γ_incoherent_ [50]. Moreover, the phase/GaAs interface energy is always the same regardless of the Ni thickness (because the phase is epitaxial on GaAs), so the activation energy ∆G* depends only on the initial interface energy of Ni/GaAs (Equation (2)). As the Ni is epitaxial (coherent interface) with GaAs substrate for the 20-nm-thick Ni in this case, the system provides more ∆G*_epitaxial_ energy to germinate a new phase and the interface energy budget is larger in this case (Equation (1)). For the incoherent interface, the germination barrier ∆G* is low, which makes the phase germination easy, thanks to the initial incoherent interface energy that minimizes the interface energy budget (γ). This corresponds to the cases of 100- and 500-nm-thick layers, explaining that the Ni_3_GaAs phase appears at 140 °C. As a result, the initial state of the interface Ni/GaAs is the reason behind the different formation temperature of the first phase.

The formation temperature of the second phase also varies with the thickness of the Ni. It is higher for the 20-nm-thick sample compared to the other two samples (Figure 3 and Figure 5). We can explain this difference by TiN/Ni interface nature. From the pole figures (Figure 2), TiN is epitaxial with Ni of 20 nm. However, no texturing for TiN was observed for the two other samples. As a result, TiN/Ni interface is coherent for the 20-nm-thick Ni sample, which explains this delay in the second phase formation. It is possible that NiAs was formed due to the change in the chemical composition of the Ni_3_GaAs ternary phase. One can assume that the ternary phase composition changes during the reaction from Ni_3_GaAs to NiAs as follows: Ni_3_GaAs → Ni_3−x_GaAs_1−x_ + xNiAs [31,47,51].

## 5. Conclusions

In conclusion, the investigation of the initial state of the as-deposited samples of the Ni/GaAs system shows that there is no intermediate phase for the three 20-, 100-, and 500-nm-thick films, but the Ni is deposited with the fcc structure and it is in epitaxy with the GaAs substrate according to the relation (001) Ni//(001) GaAs and [111] Ni//[110] GaAs for the lowest thickness of Ni while for the highest thicknesses, the Ni is no longer in epitaxy. The reaction between 100- and 500-nm-thick Ni with GaAs allows the formation of the Ni_3_GaAs phase from 140 °C; this low formation temperature can be considered as an advantage for the ohmic contacts for the targeted applications. This phase grows in epitaxy and verifies the same texture and epitaxy relationship found for the 20-nm-thick Ni film. There is no effect of the thickness on the chemical nature of the first intermetallic phase. The only difference is the formation temperature. We found that 20-nm-thick Ni starts to form at 190 °C due to the difference in the interface nature observed for Ni/GaAs when increasing the film thicknesses. We showed that the so-formed Ni_3_GaAs phase undergoes a homogeneity of the composition after the total or partial consumption of Ni film. This composition homogenization is a consequence of the thermodynamic equilibria evolution. For all thicknesses, the intermetallic phase decomposes at the end of annealing into the Ni_3−x_GaAs_1−x_ phase and the xNiAs binary phase of the same structure with the same texture as the so-formed Ni_3_GaAs phase.

## Data Availability

Not applicable.
